# Workshop report: Clinical training and integration of genetic counselors into interprofessional teams in the German-speaking countries

**DOI:** 10.1016/j.gimo.2024.101855

**Published:** 2024-05-29

**Authors:** Gunda Schwaninger, Kathrin Taxer, Sabrina Marti, Simona Cionca, Petra Freilinger, Corinne Gemperle, Anna Kalantidou, Heidi Stöhr, Sina Ramcke, Christina Wölwer, Sabine Rudnik-Schöneborn, Johannes Zschocke

**Affiliations:** 1 Institute of Human Genetics, Medical University of Innsbruck, Innsbruck, Austria; 2 Institute of Laboratory Medicine, Team Medical Genetics, Kantons-Hospital Aarau, Aarau, Switzerland; 3 Health University of Applied Sciences Tyrol, Innsbruck, Austria; 4 Genetikum, Neu-Ulm, Stuttgart, Germany; 5 Devision of Metabolism, University of Zurich, Zurich, Switzerland; 6 Institute for Human Genetics, University Clinic, Regensburg, Germany; 7 Institute for Human Genetics, University Clinic, Hamburg-Eppendorf, Hmaburg, Germany; 8 Center for Hereditary Breast and Ovarian Cancer, University Hospital Cologne, Cologne, Germany

**Keywords:** Austria, Clinical supervision, Genetic counselor, Germany, Switzerland

## Abstract

In spring 2022, the inaugural cohort of Genetic and Genomic Counseling MSc students graduated from the Medical University of Innsbruck, representing a significant milestone for the establishment of the genetic counselor (GC) profession in the German-speaking countries. A pivotal component of their education was a 15-week clinical training period. The placement experiences of both students and supervisors offered valuable insights into the attitudes of medical geneticists toward the profession. To share the knowledge gained by these pioneers and offer guidance to potential placement institutions, the MSc program team organized an online workshop on the education and training of GCs in German-speaking countries in October 2022. The first part of the workshop focused on the training program and professional profile of GCs in the German-speaking countries. It covered educational aspects, such as the structure of the MSc degree program and specific communication training. Additionally, it addressed challenges arising from the integration of GCs into existing genetics centers. The second part of the workshop delved into first-hand experiences of genetic counseling students and placement providers during clinical training. Featuring insights from supervisors, this session facilitated a discussion about the roles of GCs within interprofessional genetics teams. The workshop participants agreed on a growing demand for GCs and highlighted positive experiences, as well as challenges within participating institutes that had hosted a student. In this article we summarize suggestions for the education and training of GCs in the German-speaking countries where the profession is newly established.

## Introduction

In October 2022, the teaching team of the MSc degree program in Genetic and Genomic Counseling (GGC-MSc) at the Medical University of Innsbruck organized an online workshop on “training and supervision of genetic counselors (GCs) in the German-speaking countries,” connecting placement providers, students, graduates, and professionals in the field. The primary objective of this workshop was to foster discussion about the experiences and obstacles encountered during GC student clinical training, as well as the integration of program graduates into clinical genetics teams.

Clinical training is pivotal in preparing GC students to become proficient and empathetic health care providers capable of delivering high-quality care to individuals and families affected by genetic conditions. International experiences highlight that establishing GC training programs in a new region poses significant challenges, among them the delivery of high-quality clinical training.^1^, ^2^, ^3^, ^4^, ^5^, ^6^ These challenges include the scarcity of experienced GCs available to supervise students, limited awareness of the profession itself, and a shortage of available positions for clinical placements. Maintaining consistent quality and standards across different training sites proves difficult. Although some students may benefit from exposure to a wide array of cases and experiences, others may face limited opportunities, impacting their readiness for professional practice. Moreover, the availability and commitment of qualified supervisors is essential in ensuring adequate support for students as they develop their clinical skills and confidence. These factors are crucial for students’ professional growth and for meeting program standards for graduation.^7^ This is one reason that in Europe, other than the United States where graduates can sit the board exam right after graduation, GCs have to complete an additional 2 year trainee phase in a genetics center before they are able to register with the European Board of Medical Genetics.^8^

Therefore, the objective of the workshop was to present placement providers with a comprehensive background of the academic curriculum, insights from experienced supervisors and graduates, and to foster alignment in training standards. Additionally, the workshop aimed to clarify expectations regarding the roles and responsibilities of students during their clinical training. Here, we summarize the presentations and conclusions of the workshop, including a description of the Innsbruck GGC-MSc program. More details on the current state of GC profession in Germany, Austria, and Switzerland are provided in a separate article in this issue.

### Workshop participation and structure

Fifty-five individuals participated in the workshop, including 22 medical geneticists (representing 21 university and privately managed genetic centers from Germany, Austria, and Switzerland, the D-A-CH region), 1 senior GC from the United Kingdom, 8 GGC-MSc graduates from Germany and Austria, 10 GC students, 12 prospective students, and 2 participants from other related professions. The genetic centers represented in the workshop had either already provided clinical training for GC students or expressed interest in doing so. The first author corresponded with both speakers and participants before the workshop and documented their online presence in an Excel sheet.

The half-day workshop encompassed 7 presentations and an open discussion regarding the integration of GCs into clinical genetics teams (see program in Supplemental Figure 1). The Innsbruck program team conceptualized, moderated, and facilitated the workshop. Participants were informed that presentations would be recorded. The presentation segment used a classic frontal methodology, with the GGC-MSc program team laying the knowledge foundation and placement providers and GGC-MSc graduates offering first-hand accounts of their training experiences. This was followed by an interactive format that allowed all participants to voice questions, opinions, and proposals for GC training and integration. All presentations were video recorded and summarized to create the original workshop report, forming the basis for this manuscript. The discussion was not video recorded to ensure an open exchange of possibly controversial opinions but was documented in writing to capture key points. A preliminary report was shared with all coauthors and several speakers for validation.

### Background for clinical supervisors

#### University training for GCs

The curriculum was presented by the first Austrian registered GC and MSc program director in Innsbruck. The first 2 semesters cover a comprehensive blend of theoretical aspects in genetic counseling concepts, genetics, genomics, diagnostics, risk calculation and statistics, and communication and practical counseling skills (Box 1). This “consolidation” phase enables students from diverse academic backgrounds to acquire fundamental GC knowledge. Genetics and communication training are given equal emphasis and carry a similar weight in terms of credits. Although the classroom setting offers opportunities to practice patient-centered counseling techniques across various scenarios, practical training at clinical placements is indispensable to learn from real-life patient interactions.

Box 1 **Curriculum of the GGC-MSc (120 ECTS credits-European Credit Transfer System)**

**Table:** 

- KON Concepts, principles, questions and interventions in genetic counseling (5 ECTS)
- GEN Human Genetics, medical genetics and genomics (7 ECTS)
- GEV Structure of the healthcare systems & interprofessional cooperation (5 ECTS)
- LAB Genetic and genomic diagnostics and bioinformatics (4 ECTS)
- KOM 1-2 Counseling and communication (11 ECTS)
- STA Probability calculation and statistics in medical genetics (3 ECTS)
- COP 1-3 Applied Genetic Counseling 1-3 (15 ECTS)
- ERK Ethical, legal and cultural aspects in medical genetics (7 ECTS)
- PRK Genetic counseling placements (36 ECTS)
- WIS Research methods (7 ECTS)
- MAS Master thesis & defense (20 ECTS)

The second to fifth semesters of the program feature a seminar series named “Applied Genetic Counseling (COP)” integrating the genetic counseling theories, psychosocial understanding, and counseling skills acquired in the first year with focused teaching blocks that address the most prevalent and essential genetic disorders encountered by GCs throughout their professional careers. Through case discussions and role plays, the biomedical, psychosocial, and communication aspects of these conditions are explored and practiced in collaboration with a multiprofessional teaching team. Additionally, regular group supervision sessions are conducted to facilitate case management and delineation of tasks within the multiprofessional team (Box 2).

Box 2 **The modules “Applied Genetic Counseling COP 1-3” are taught from the 2nd to the 5th semester and follow the structure of disease blocks. Teaching includes the following:**

**Table:** 

- Scientific knowledge about genetic conditions
- Clinics, diagnostics and therapy for important genetic diseases
- Therapy measures, check-ups and patient advocacy groups
- Research of background information based on literature databases
- Genetic counseling approaches to diagnostic questions
- Preparation of consultation letters
- Presentations and case discussions on genetic conditions
- Counseling scenarios/ case analysis
- Balint groups and supervision
- Written reflection on cases
- Understanding of the particular ethical challenges of genetic medicine
- Working according to the international code of practice and code of ethics for GCs
- Knowing the relevant legal aspects of genetic medicine
- Applying internal, national and international protocols, directives and guidelines
- Being aware of one’s professional competencies and limitations as a GC

Starting in the second semester, COP first focuses on reproductive medicine and onco-genetics, aiming to prepare students for the first challenges at their placements. From the third to the fifth semester, the discussions delve into more advanced indications (Supplemental Box 1), encompassing a wide range of genetic conditions, such as metabolic disorders, internal disorders, neurological disorders, connective tissue disorders, prenatal and predictive testing, congenital malformations and genetic syndromes, hearing and visual impairments, mitochondrial diseases, non-Mendelian inherited disorders, variants of uncertain significance, and incidental/secondary findings. Throughout the second year of the program, students also acquire knowledge on the ethical and legal foundations of the GC profession. Furthermore, the necessary skills to independently conduct a research project for their master’s thesis are acquired.

#### Standardization of placements for GC students

The clinical placement in a genetic service center accounts for 36 European Credit Transfer System credits (equivalent to 75 days of 8 hours each). In the first semester of the MSc program, a placement agreement is signed between the student and the placement institution that sets the timely allocation of the internship and serves as a formal agreement on what is expected from the student and the placement provider. The allocation of the 75-day placement period between the second and fourth semesters is flexible, allowing for completion in 1 or multiple blocks or on specific days of the week. Placements in multiple centers are encouraged to provide students with a diverse range of experiences in the field of medical genetics. Additionally, we foster international rotations (eg, in centers in French-speaking Switzerland and the United Kingdom) to give students insights into the work of GCs internationally.

**Figure 1:**
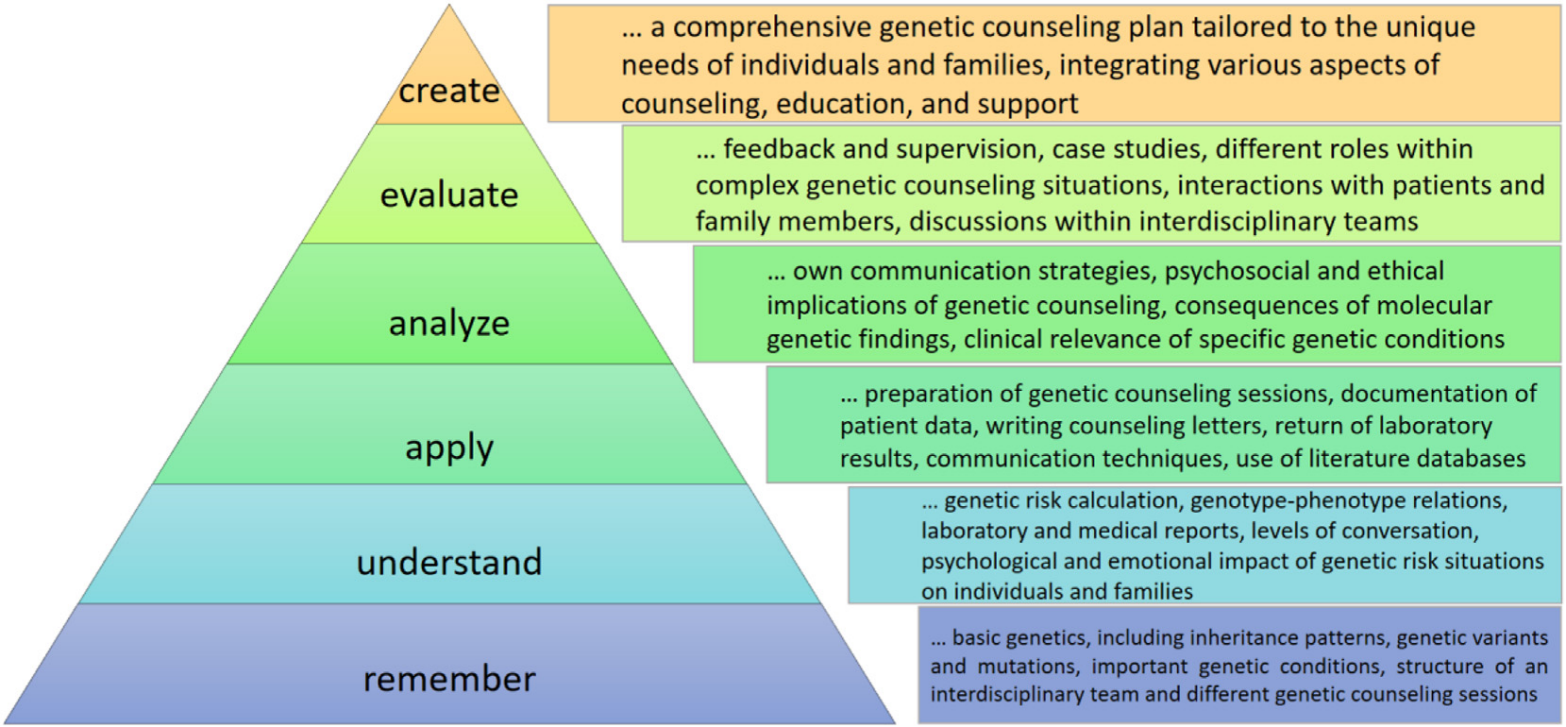
**Blooms taxonomy applied to genetic counseling training.**^9^

A senior clinical geneticist and MSc program team member in Innsbruck, reported how GCs are integrated into the workflow of a clinical genetic service in Austria, where all clinical indications are seen, and a broad range of cytogenetic and molecular genetic laboratory methods are offered. The training of genetic counseling skills follows the taxonomy developed by Benjamin Bloom.^9^ This taxonomy is designed to help educators build the different levels of cognitive skills from recalling foundational knowledge to creating and implementing advanced counseling strategies. This approach ensures a comprehensive and developmental learning experience for future GCs (Figure 1).

At the beginning, students observe and listen in to remember and understand the different tasks of genetic counseling and the structure of the consultation. Further into the placement, they are actively integrated into in the counseling process, applying, analyzing, and evaluating their skills. Students are expected to participate in at least 80 genetic counseling cases, documenting their experiences. The acquired skills of the students should be closely monitored throughout the placement, ensuring that a balanced level of involvement in various aspects, such as consultation preparation and documentation, active participation in a growing part of the counseling session, and composition of consultation letters. Students are expected to prepare for each case by gathering the patients’ medical history and the natural history of the disease in question, discussing any uncertainties with their supervisor before the consultation. During the consultation, students new to the placement are encouraged to document patient and family history. Further along students should also contribute to patient education including discussion of the natural history of hereditary diseases, inheritance patterns, recurrence risk, and testing options, also addressing psychosocial concerns. During the placement, students are expected to actively seek feedback and exchange ideas with their supervisors.

#### Placement feedback and supervision

To facilitate a structured feedback and supervision process during clinical training, the GGC-MSc team requires students to maintain placement documentation. This includes a case log that adheres to the specifications of the European Board of Medical Genetics (EBMG) registration system, an attendance sheet, feedback forms for the half-time and final assessment, and a written reflection on the placement experience. In the workshop, a medical geneticist and MSc program team member, summarized the intense communication training to prepare prospective GCs for different levels of genetic consultations. During this theoretical and practical training, communication experts provide students with a skill set for patient interaction and encourage its application in role plays. To foster communication and clinical competencies during placement, feedback on the students’ performance should be provided immediately after each counseling session and more formally in 2 written assessments. An anonymized case diary is utilized for self-reflection, case studies, counseling group supervision, and writing a reflective piece. By the end of the placement, students should have conducted a minimum of 10 complete genetic counseling sessions under supervision, including documentation and consultation letters. Half of these consultations should focus on onco-genetics and the remaining half on other conditions. Ideally, students under supervision should have also communicated at least 3 “bad news results” to patients. After the final feedback, the supervisors determine whether the clinical team regards the student proficient to continue training in accordance with the EBMG Code of Professional Practice. Currently, most of our clinical supervisors are medical geneticists because senior GCs are still rare within the German-speaking region.

### Experiences with GCs in different clinical settings

The workshop revealed both growing acceptance and challenges of the early integration phase of the GC profession in interprofessional teams in genetic centers in the D-A-CH region. Experiences from GGC-MSc graduates and medical geneticists were discussed.

#### Expectations by placement providers

An active GC and graduate of the first Innsbruck MSc program, reported the results of structured interviews with placement supervisors on their experience with the first GC students. Her study effectively captured the attitudes of placement providers toward the skills and future relevance of GCs in German-speaking genetic services. All 7 students successfully performed the necessary tasks within the interdisciplinary teams as expected by their placement supervisors. Tasks included consultation preparation, participation in educating and counseling patients, risk calculation, and writing consultation letters, all under supervision. The applicability of the program curriculum was emphasized, and the successful integration of students was confirmed by the placement providers. Most notably, the supervisors did not anticipate a loss in counseling quality compared with medical geneticists and estimated an increase in counseling efficiency by up to 50% for a medical geneticist working with a GC student. Challenges raised in the interviews included the legal situation of GCs, remuneration concepts, and supervisor time expenditure for the training.^10^

#### Experiences with GCs in a university genetics center

A medical geneticist and a GC graduate from the Institute of Human Genetics at the University of Hannover jointly presented on how they integrated a GC into a clinical genetics team. Several team members had prior experiences with GCs from working abroad and implemented a “tandem approach” by which the GC started the consultations alone and was then joined by a medical geneticist. Depending on the consultation type and the experience level, the GC gradually gained autonomy. A medical geneticist always joined toward the end for informed consent and complex patient questions. The GC wrote drafts of consultation letters and delivered test results with the medical geneticist present. Challenges included supervision time and adjusting processes for the new profession. Having defined GC skills at each training stage was seen as beneficial. Future suggestions included that GCs could establish interprofessional networks, serve as primary patient contacts, and assist in coordinating patient care. Overall, the presentation highlighted increased consultation quantity and quality and better team dynamics.

#### Experiences with GCs in a privately managed genetics center

A medical geneticist and placement supervisor from a privately managed genetics center, MGZ Munich, shared insights into the daily routine when working with GCs. Alongside a student from Innsbruck, 2 German-speaking GC students from the United States also completed their placements in this service. Early on in their training, GCs started to assess patient family history and gradually took on more responsibilities, such as goal setting, leading through the consultation, providing oral and written information, writing consultation letters, communicating findings in collaboration with the medical geneticist, and serving as primary patient contacts. The presentation highlighted the GCs’ communication skills, the calm atmosphere, and expansion of counseling capacities that allowed physicians to focus on medical tasks such as differential diagnoses and individual treatment strategies. It was proposed that GCs could specialize in various genetic fields and connect with support groups to improve patient benefits. Challenges faced are the lack of GC regulation and billing for GC services, particularly affecting nonpublicly managed centers.

#### Workshop open discussion

During the workshop discussion, some possible obstacles for the implementation of GCs in German-speaking countries were addressed. Mostly professional issues were raised, such as the scope of practice, remuneration, billing, and regulation (further addressed in a second article in this issue). Medical geneticists agreed that the GC students needed considerable time for supervision because GCs were from different nonmedical backgrounds. It was recommended that GCs introduce themselves with their equivalent German professional title “Genetische FachberaterInnen” at the beginning of the session, shortly explaining their profession and communicating that a physician would join further along the consultation. As already outlined in a previous workshop, there should be a clear agreement about tasks that are and that are not taken over by the GC.^11^ It is also common for GCs to provide patients with personal business cards, offering a convenient way to contact them directly for further inquiries. Several workshop participants observed that patients perceive GCs as more approachable than medical doctors and may feel more at ease in asking questions. The unique training of GCs was seen as a clear advantage in the counseling setting. To foster the positive interprofessional cooperation and optimize patient care, it is crucial for medical geneticists and GCs to work together closely and build trust in their professional relationship.^12^ Both professions make valuable contributions to patient care,^13^ and considering the growing demand for genetic testing and counseling, the integration of GCs was widely regarded as helpful by workshop participants. The need for an increased number of trained GCs was expressed and the implementation of additional training programs for GCs in the D-A-CH region suggested.

### Conclusion

The workshop highlighted the increasing demand for GCs in the German-speaking countries. Nonetheless, the implementation of clinical training for GCs is a huge challenge in countries in which the GC profession is newly established. There are hardly any senior GCs to learn from, and most medical geneticists have little or no experience with GCs unless they trained abroad. This necessitates a close interaction of the GGC-MSc team in Innsbruck with placement providers. Detailed documents are provided to harmonize clinical training. Regular workshops help to inform partners about the new profession and to communicate expectations from both the GC students and their supervisors. The overall perspective on the integration of GCs has undergone a positive shift since the initiation of the GGC-MSc in 2019. To foster successful collaboration within multiprofessional genetics teams, it is imperative for GCs and medical geneticists to unite and work together for the positive development of the field.

## Data Availability

The corresponding author confirms that she has full access to all the data in the study and takes responsibility for the integrity and the accuracy of the data. Presentations and excerpts of the discussions in the course of the workshop presented in this article are available upon request from the corresponding author.

## Declaration of AI and AI-Assisted Technologies in the Writing Process

During the preparation of this work the first author used ChatGPT to check for language, spelling, and grammar. After using this tool, the first author reviewed and edited the content as needed and takes full responsibility for the content of the publication.

## Conflict of Interest

Kathrin Taxer, Sabrina Marti, Simona Cionca, Petra Freilinger, Corinne Gemperle, Anna Kalantidou, Heidi Stöhr, Sina Ramcke, and Christina Wölwer are current graduates of the MSc program in Genetic and Genomic Counseling at the Medical University of Innsbruck. Gunda Schwaninger, Sabine Rudnik-Schöneborn, and Johannes Zschocke are program director and part of the development and teaching team of the master program in Genetic and Genomic Counseling at the Medical University of Innsbruck.

## Additional Information

Supplementary Figure 1 and Supplementary Box
