# Supplementary material for: Workshop report: Clinical training and integration of genetic counselors into interprofessional teams in the German-speaking countries
Source: Genet Med Open. 2024 May 29;2(Suppl 2):101855. doi: 10.1016/j.gimo.2024.101855 (PMC11658553; doi:10.1016/j.gimo.2024.101855)
Supplement: Supplementary Figure 1 and Supplementary Box [file mmc1.docx]

Supplementary Figure 1. Program of the Workshop on training and supervision of genetic counselors in the German-speaking countries.


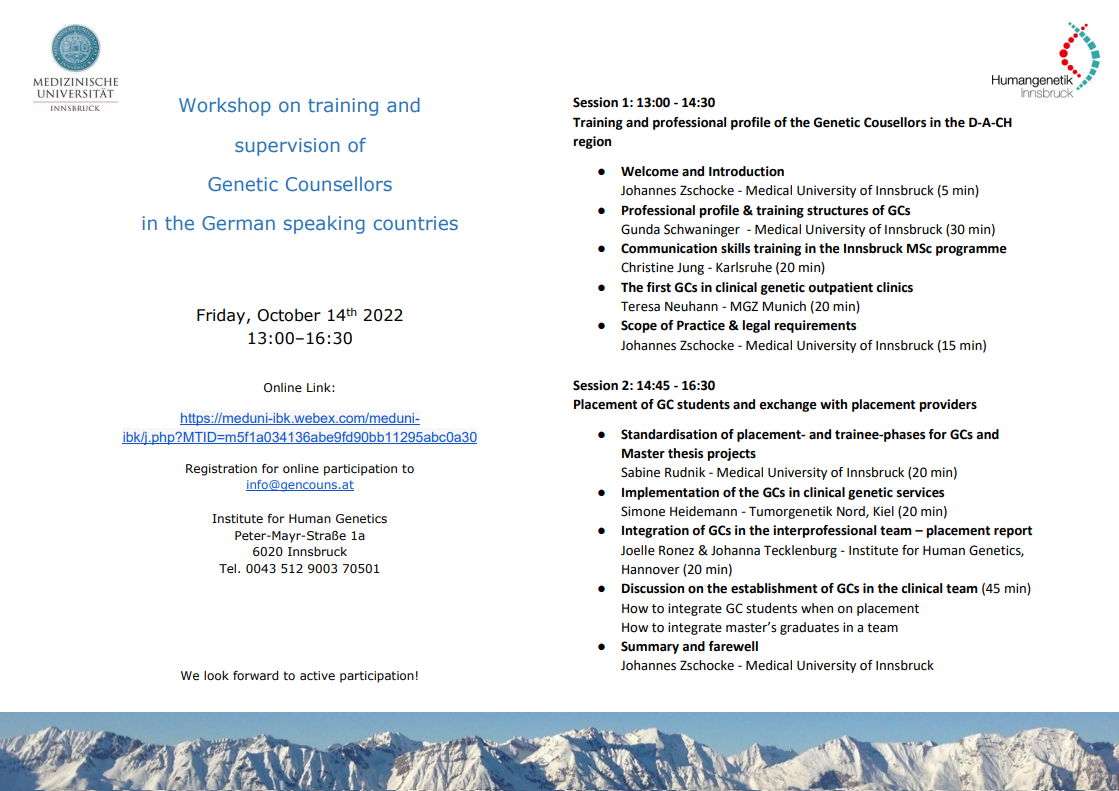


Supplementary Box 1. Tasks that need to be trained during clinical placement

- Active participation in genetic consultations
- Participation in genetic case discussions, seminars and meetings in the institute and with other clinics
- Learning and practicing patient-centered communication of the complex biology of genetic disorders
- Preparation and follow-up of cases, including administrative tasks during genetic consultations
- Case discussion with colleagues in case of ambiguity and for case reflection
- Competent compilation of the personal history of individuals in the context of clinical-genetics questions
- Systematic recording and standardized documentation of the family tree
- Use of human genetic databases for diagnosis and management of genetic diseases
- Understanding of the complexity of onco-genetic issues
- Risk calculation for genetic disease; risks for patients, offspring and relatives
- Learning and practicing non-directive genetic counseling regarding individual options available in the event of personal or family health risks, especially with regard to possible genetic diagnostics
- Communicating and interacting professionally with all patients (children and young people and their families with and without intellectual disabilities)
- Learning and practicing the indications and interpretation of genetic laboratory analyses
- Genetic counseling before and after genetic laboratory analyses with special attention to the requirements of the genetics laws under medico-legal supervision by a specialist in medical genetics
- Profound knowledge of the 30 most important genetic diseases (textbook compilation)
- Knowledge of the special issues in rare genetic diseases
- Writing clinical-genetic consultation letters
- Presenting cases in clinical meetings
- Psychosocial care of patients and family members and patient support (empowerment)
- Referring to additional psychological care if needed
- Connecting to patient advocacy groups and patient representatives
- Educating patients to improve informed consent
